# Cell-cell adhesion interface: rise of the lateral membrane

**DOI:** 10.12688/f1000research.10680.1

**Published:** 2017-03-15

**Authors:** Vivian Tang

**Affiliations:** 1 Department of Cell and Developmental Biology, University of Illinois, Urbana-Champaign, IL, 61801, USA

**Keywords:** lateral membrane, wound healing, regeneration, acto-myosin, cell-cell adhesion, epithelial tissue

## Abstract

The lateral membrane plays an important role in the mechanical stability of epithelial cell sheet in steady state. In addition, the lateral membrane is continuously remodeled during dynamic processes such as cell extrusion, cytokinesis, and intercellular cell movement. In wound healing, the lateral membrane must be built from flat and spread cells that had crawled into the area of the wound. Thus, forming the lateral membrane is a phenomenon that occurs not only in development but also during homeostatic maintenance and regeneration of differentiated epithelial tissues.

## Introduction

An epithelial cell consists of three plasma membrane regions: the apical, the lateral, and the basal membranes ^1^. These three plasma membrane domains have distinct lipids and proteins and thus perform different functions. The apical plasma membrane is a free surface that is exposed to the luminal fluid. The basal plasma membrane is attached to an extracellular matrix that supports the epithelial tissue. The lateral membrane of the epithelial cell orients perpendicularly to the apical and basal membranes and frequently is referred to as cell-cell contacts, the cell boundary, or intercellular junction. Depending on whether the native epithelium is squamous, cuboidal, or columnar, the area of the lateral membrane can range from about 10% to 60% of the total cell surface area.

The lateral membrane contains proteins for cell-cell adhesion, intercellular signaling, and cell-cell communication and is the only region of the plasma membrane where an epithelial cell interacts with other epithelial cells. The relationship between the lateral membrane and intercellular interaction is especially important for non-cell-autonomous processes such as mechano-regulation of cell-cell adhesion. By providing an interface for homophilic interactions between adhesion molecules such as E-cadherin, the lateral membrane allows neighboring cells to push and pull on adhesion complexes from the outside of the cell. Interactive mechanical regulation of cell-cell adhesion by the direct actions of the neighboring cells can be achieved only when cell-cell adhesion molecules are positioned on the intercellular interface. Hence, the lateral membrane plays a permissive role in the strengthening of cell-cell adhesion and the maturation of adhesion complexes.

**Figure 1.**
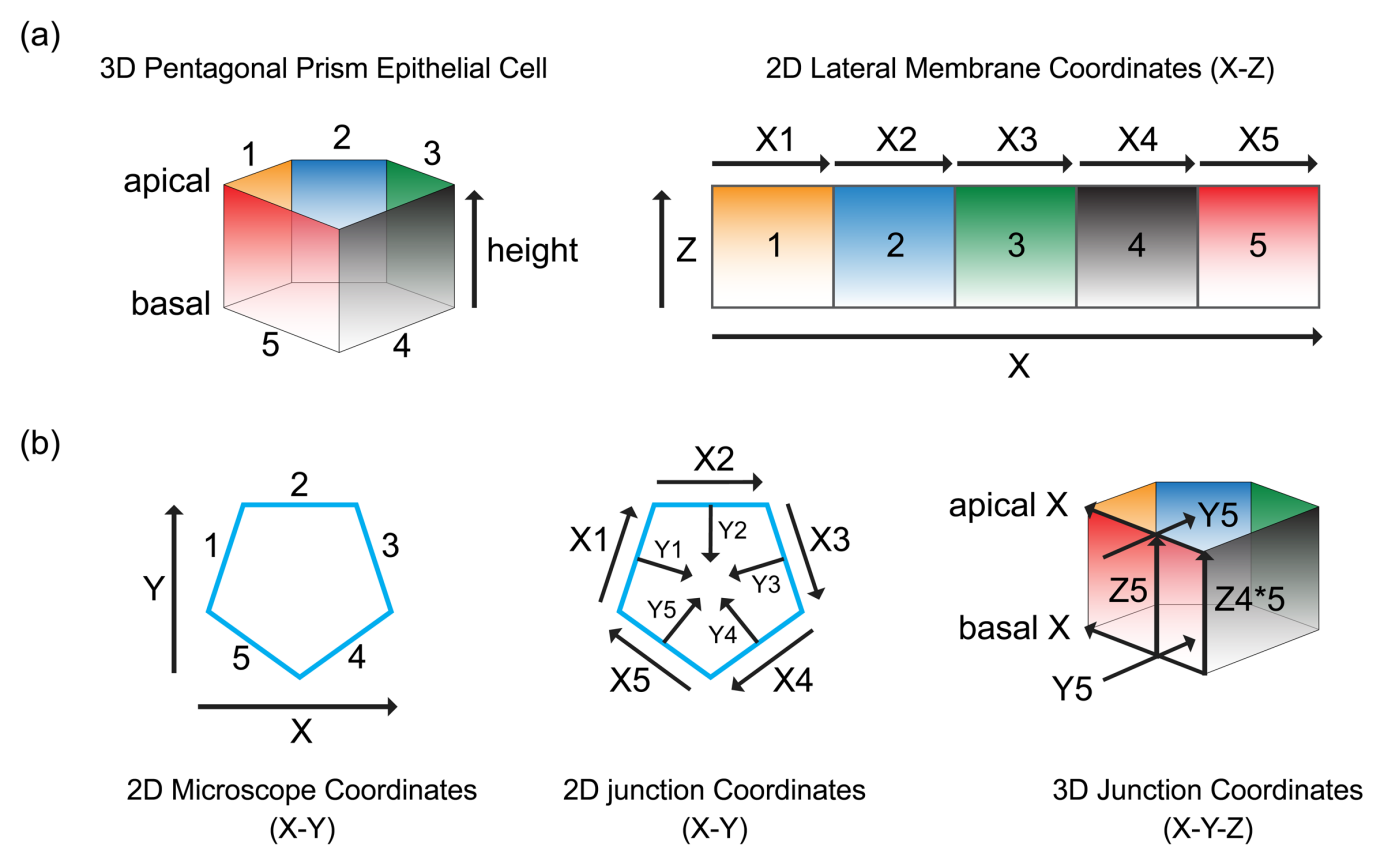
Epithelial lateral membrane is a three-dimensional structure. ( **a**) The lateral membrane of an epithelial cell forms distinct interfaces, 1–5, with different neighboring cells. The cell-cell boundaries form the cell junctions, X1–X5, representing the X-axis of the lateral membrane. A gradient of proteins can be found along the Z-axis of the lateral membrane, from apical to basal membrane. ( **b**) The Y-axis of the lateral membrane and cell junction is perpendicular to the X-axis. The X- and Y-axis of the lateral membrane and cell junction are different from the microscope X-and Y-axis. The X- and Y-axis of the cell junction remain the same along the Z-axis only if the epithelial cell is a perfect prism.

The lateral membrane of an epithelial cell can take on a different identity when interacting with different neighboring cells, resulting in the emergence of separate and independent lateral membrane domains ( Figure 1). The lateral plasma membrane of vertebrate epithelial cells can be functionally and structurally divided into the upper, middle, and lower regions on the basis of differential distribution of membrane proteins. The upper lateral membrane lies immediately adjacent to the apical membrane. The upper lateral membrane contains the tight junction, the adherens junction, and the gap junctions, collectively known as the apical junction ^2^. The middle part of the lateral plasma membrane contains the desmosomes and the lateral adherens junctions ^3^. The lower lateral plasma membrane lies immediately adjacent to the basal membrane and contains the basal adherens junction ^4^ and protrusive structures known as cryptic lamellipodia ^5^. Cell adhesion proteins are frequently concentrated at the apical junction but also distributed along the entire surface of the lateral membrane. Adhesion proteins found on the middle and basal regions of the lateral membrane are not co-localized to the same extent as when they are on the apical junction ^6– 11^. Indeed, the strength of cell-cell adhesion and acto-myosin activities forms a gradient along the vertical axis of the lateral membrane ^12, 13^. Hence, the lateral membrane consists of functionally distinct vertical slices with different neighbors distinguishing their identities and horizontal slices with different adhesion complexes distinguishing their properties. The lateral membrane forms a hollow cylinder that houses the cytoplasm and thus contains both two-dimensional information on the X-Z plane ( Figure 1a) and three-dimensional geometric and force information along the Y-axis ( Figure 1b).

**Figure 2.**
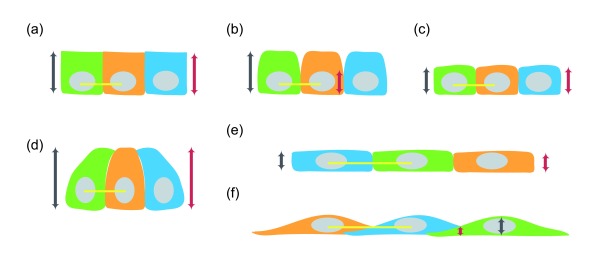
Phenotypes of abnormal formation of the lateral membrane. ( **a**) The height of wild-type epithelial cell (grey line with arrowheads) is the same as the height of its lateral membrane (red line with arrowheads). ( **b**) Decrease in the height of the lateral membrane (red line with arrowheads) without a change in the height of the epithelial cell (grey line with arrowheads). ( **c**) Decrease in both the height of the lateral membrane (red line with arrowheads) and the height of the epithelial cell (grey line with arrowheads) with the same cell diameter (yellow line) as wild-type cells. ( **d**) Increase in both the height of the lateral membrane (red line with arrowheads) and the height of the epithelial cell (grey line with arrowheads) with the same cell diameter (yellow line) as wild-type cells. ( **e**) Decrease in the height of the lateral membrane (red line with arrowheads) and the height of the epithelial cell (grey line with arrowheads) with increase in cell diameter (yellow lines mark twofold change). The lateral membrane remains vertically positioned. ( **f**) Decrease in Z-height of the cell-cell interface (red line with arrowheads) can result from formation of orthogonal lateral membrane, leading to decrease in cell height (grey line with arrowheads) with increase in cell diameter (yellow lines mark twofold change).

**Figure 3.**
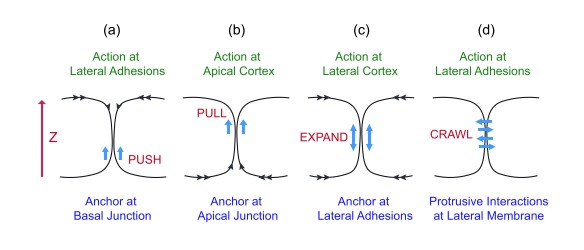
One-dimensional treatment of lateral membrane formation. ( **a**) Pushing from the bottom of the lateral membrane. ( **b**) Pulling from the top of the lateral membrane. ( **c**) Expansion toward both top and bottom from the middle of the lateral membrane. ( **d**) Crawling along the lateral membrane of neighboring cell.

The purposes of this commentary are to briefly summarize recently published phenotypes associated with abnormal formation of the lateral membrane ( Figure 2a–f) and to discuss possible mechanisms that help create this intercellular interface in epithelial cells ( Figure 3– Figure 6).

**Figure 4.**
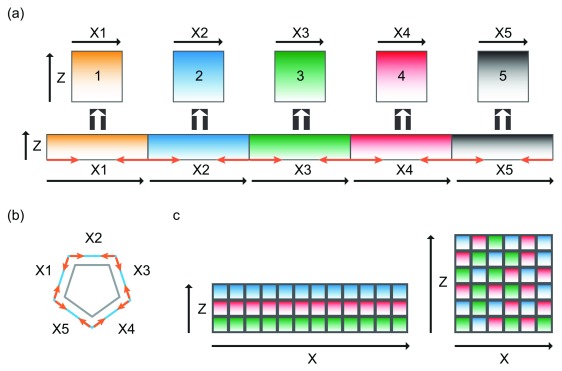
Two-dimensional treatment of lateral membrane formation. ( **a**) Shrinking of the lateral membrane on X-axis results in an increase in lateral membrane height on the Z-axis and a decrease in the length of the X-axis. Orange arrows represent direction of shrinkage. ( **b**) Decrease in the length of X-axis (outer blue pentagon) results in a smaller diameter of the cell (inner grey pentagon). Orange arrows represent direction of shrinkage. ( **c**) The lateral membrane is represented as a collage of individual membrane domains (small squares). In flat cells, there are more squares forming the X-axis than the Y-axis (left collage). Increase in the height of the lateral membrane represented by a re-shuffling of the individual membrane domains, resulting in increase in the Z-length and decrease in the X-length (right collage).

**Figure 5.**
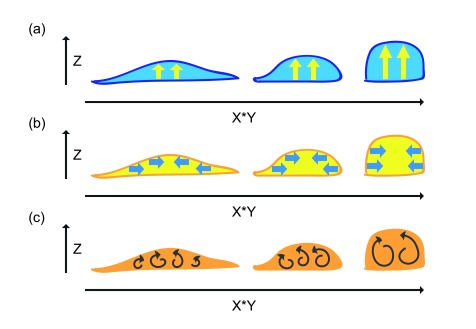
Three-dimensional treatment of lateral membrane formation requires coupling the plasma membrane to the cytoplasm. ( **a**) Protrusion of the cytoplasm forces re-shaping of the cell surface to create the lateral membrane. ( **b**) Contraction of the cytoplasm forces re-shaping of cell surface to create the lateral membrane. ( **c**) Cytoplasmic flow generates thrusting force in the cytoplasm to produce cell shape changes.

**Figure 6.**
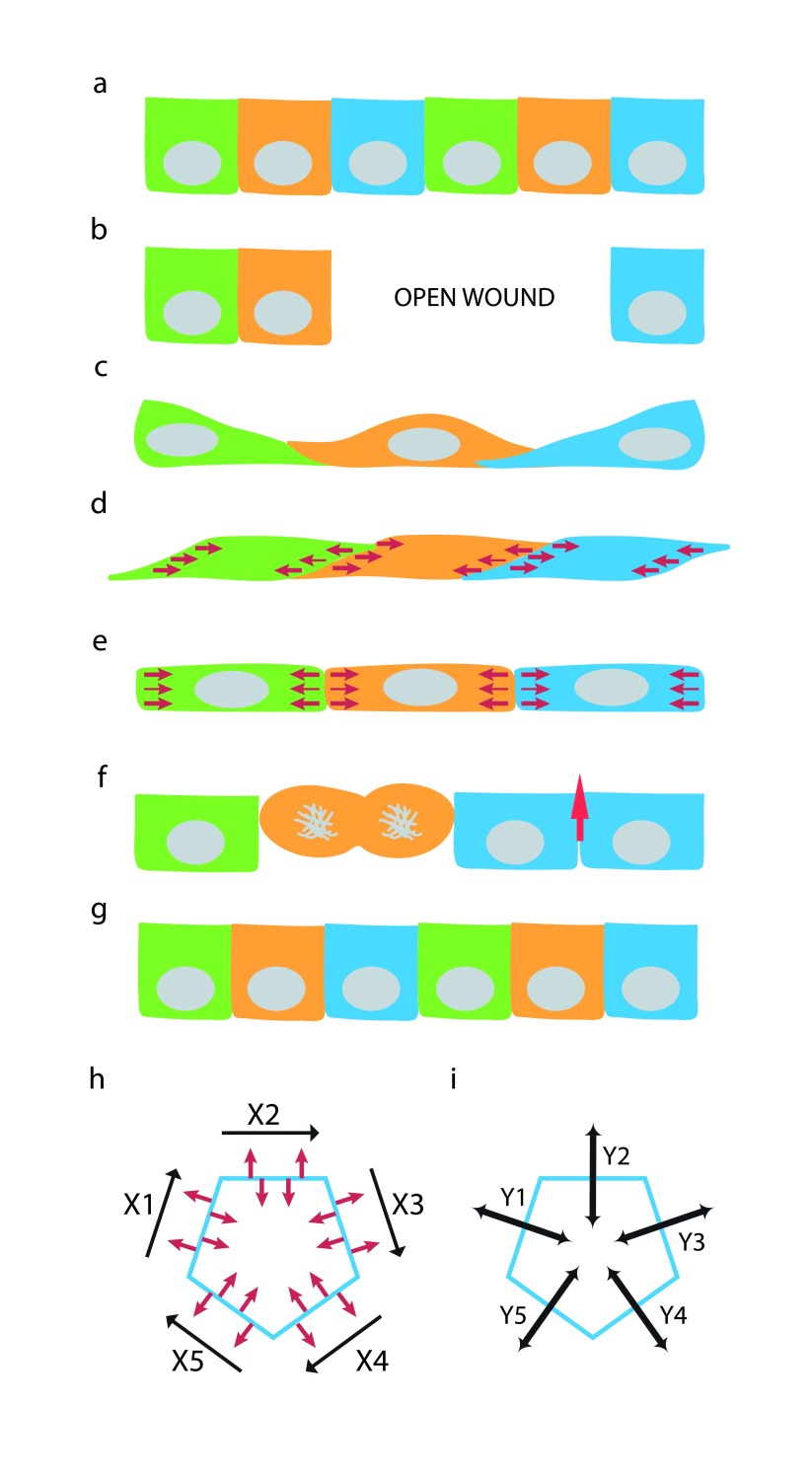
Generation of the vertical lateral membrane from orthogonal lateral membrane. ( **a**) Steady-state monolayer of epithelial cells with vertically positioned “upright” lateral membrane. ( **b**) Wounding an epithelial monolayer leaves an open area. ( **c**) Epithelial cells migrates to cover the open area. Orthogonally positioned “slouching” lateral membrane is formed between spread cells that had just migrated into the wound. ( **d**) Force exerted along the lateral membrane and on the Y-axis of cell-cell interface realigns the slouching lateral membrane. ( **e**) Balancing the forces from apposing cells along the Z-axis can ‘verticularize” the lateral membrane to form an upright cell-cell interface. ( **f**) Restitution of epithelial layer by re-population with new cells via cell division and elongation of vertical intercellular interface (red arrow). ( **g**) Completion of restitution result in steady-state monolayer with vertical Z-axis once again. ( **h**) Conversion of orthogonal lateral membrane to vertical lateral membrane in ( **d**) and ( **e**) can be achieved by contraction forces along the X-axis of cell-cell junction between neighboring cells. ( **i**) Independent contraction forces can be delivered separately along the Y-axes (Y1-5) to each cell-cell interfaces (X1-5) between distinct neighboring cell-pairs. Optimal force balance at cell-cell interface depends on the epithelial ensemble and force distribution among individual cells in the epithelial monolayer.

## Lateral membrane phenotypes

Recent studies have shown that the generation of the epithelial cell-cell interface is dependent on many factors, including junctional and non-junctional proteins ^14– 28^. Disrupting the functions of these junctional and non-junctional determinants results in not only abnormal formation of the lateral membrane but also alteration of the overall cellular geometry ( Figure 2a–f). Often, the ratio of apical, lateral, and basal cell surface areas is dramatically changed, especially in cells where the lateral cell surface normally represents a significant portion of the plasma membrane. In most cases, the vertical distance between the apical and basal membrane is reduced, resulting in an overall reduction in cell height (that is, shorter cells) ( Figure 2c, e, f). One group of shorter cells has a cell diameter similar to that of wild-type cells ^14, 15^. These cells have an overall reduction in cell volume and surface area (that is, smaller cells) ( Figure 2c, yellow line marks cell diameter). The second group of shorter cells is associated with increased spread area ^16– 23^, but the overall cell volume is roughly conserved (that is, flat and large cells) ( Figure 2e–f). A variant of the shorter lateral membrane phenotype is associated with a change in the position of the apical junctional complex on the vertical axis ^24, 25, 29^. In this variant, the distance between the apical and basal cell-cell junction is shortened but the height of the cell remains the same ( Figure 2b). Another exception to the common flat and large cell phenotype is an expansion of the lateral membrane ^26, 27^, resulting in an overall increase in the height of the epithelial cells ( Figure 2d). Yet the majority of the lateral membrane defects described in the literature are associated with large and flat cells ( Figure 2e–f, yellow lines show where the diameter is twice that of wild-type cells). For this reason, this commentary will consider the problem of building the lateral membrane to be a problem of converting a flat and large cell to a taller and thinner cell—in essence, a geometry problem.

Four categories of proteins appear to play important roles in converting a spread cell to a taller cell: (1) structural components that support integrity of the lateral membrane ^19– 21^, (2) cytoskeletal dynamics on the lateral membrane ^14, 15, 18, 22^, (3) cell-cell adhesion molecules ^16, 17, 23, 30^, and (4) acto-myosin contractile activities ^14, 21, 24– 26^. In addition, some earlier studies indicate that mechanical properties of the extracellular matrix also affect generation of epithelial cell height ^25, 28^, indicating that the basal cell surface can contribute to the regulation of the lateral cell-cell interface. Disrupting the function of any one of these components leads to defective formation and shortening of the lateral membrane on the vertical Z-axis. Therefore, generating the lateral membrane is dominated not by a single set of criteria but by multiple components on the plasma membrane, the sub-membrane cortex, and acto-myosin activities inside the cell. These factors must work together, most likely in a spatially and temporally coordinated fashion, to generate the lateral membrane. However, knowing the diversity of these factors not only reveals the complexity of epithelial cell-cell interface but compels us to evaluate our understanding of the formation of the lateral membrane.

## Rise of the lateral membrane

Conceptually, the rise of the lateral membrane would require factors acting on the X, Y, and Z axes of the cell junction ( Figure 1). Three distinct aspects of the lateral membrane can be derived from the axes: (a) the height of the cell (that is, the vertical distance between apical and basal surfaces that is represented by the one-dimensional Z axis), (b) the area of the lateral plasma membrane (that is, the cell-cell interface that is represented by the two-dimensional X-Z axis), and (c) the volume surrounded by the lateral membrane (that is, the cytoplasm that is represented by the three-dimensional X-Y-Z axis). Thus, the rise of the lateral membrane is an amalgamation of factors that determine the Z height, the X-Z area, and the X-Y-Z volume. We will treat the one-dimensional, two-dimensional, and three-dimensional problems separately ( Figure 3– Figure 6). This reductionist approach, though unrealistically simplified, outlines the potential parameters that an epithelial cell might employ to create the eventual cell-cell interface. However, this commentary will not discuss the molecular players that might be involved in the generation of the lateral membrane, which has been summarized in recent reviews ^31– 35^, nor will this commentary discuss any phenotypes characterized by gross disruption in the organization of the epithelial cell layer ^26, 36^.

A one-dimensional analysis for lateral membrane expansion can be considered a zipper problem on the Z-axis ( Figure 3). It requires upward expansion of the intercellular interface between neighboring flat cells to generate taller cells. The lateral membrane can rise up by a pushing force that has an anchor at the basal plane ( Figure 3a). Alternatively, the lateral membrane can be dragged up by a pulling force that has an anchor at the apical plane ( Figure 3b). The lateral membrane can expand bi-directionally, nucleating from cell-cell attachment sites ( Figure 3c). Lastly, the lateral plasma membrane could be generated when neighboring cells send protrusions to crawl up each other ( Figure 3d). Such one-dimensional expansion of the lateral membrane can conceivably be contributed by one-dimensional biochemical processes such as actin elongation or directional movement of molecular motors ^37– 39^.

A two-dimensional analysis for lateral membrane expansion can be considered a reshaping problem on the X-Z axis ( Figure 4). The process must involve re-arrangement of lateral plasma membrane subdomains, which requires elongation of the lateral membrane on the vertical apical-basal axis and shrinking of the lateral membrane on the horizontal axis. One possibility is to squeeze the lateral membrane horizontally from the base of the cell such that excess lateral membrane would move up vertically on the apical-basal axis. This could possibly be achieved by contraction on the plane of the lateral membrane surrounding the base of the cell ( Figure 4a). Alternatively, the entire plasma membrane can be re-shuffled ( Figure 4b). This process requires the lateral membrane to consist of many individual subdomains that behave independently of each other ^40^. Re-arrangement of membrane subdomains could conceivably be driven by a boundary-based mechanism or a network-based mechanism, analogous to cell intercalation during morphogenetic events ^41, 42^.

A three-dimensional analysis for lateral membrane expansion can be considered a volume problem ( Figure 5). If the cytoplasm of the three-dimensional cell is changed from being a flat cell to being a tall cell, the plasma membrane may not have to play an active role in this process but simply to behave as a passive component like a cloth that drapes over the surface of the cytoplasm. Therefore, the generation of the lateral membrane can be discussed as a problem of cell elongation. Upward protrusion of the cytoplasm in the apical-basal axis can lead to vertical extension, resulting in an increase in cell height and consequently an increase in the height of the lateral plasma membrane ( Figure 5a). Alternatively, centripetal contraction on the horizontal axis can squeeze the cytoplasm and force the cytoplasm to elongate upward, resulting in a decrease in cell diameter, an increase in cell height, and expansion of the lateral plasma membrane ( Figure 5b). Moreover, a more dynamic and labile mechanism involving cytoplasmic flow ^43– 46^ would support force production and bulk cell shape changes ( Figure 5c). These three-dimensional processes are likely to require dynamic attachment of the cells to the extracellular matrix to provide traction during force production as well as coupling of the cytoplasm to the plasma membrane to allow force transmission to cell-cell adhesions.

However, in real life, spread cells that have migrated into a free surface, such as an open wound, often overlap with their neighbors by crawling over the top or extending protrusions underneath each other, forming an orthogonal lateral membrane that contains cell-cell adhesion proteins ( Figure 6a–c). Thus, the generation of the lateral membrane from these overlapping cells would require simply to straighten them up, by converting an orthogonal “slouching” lateral membrane to a vertical “upright” lateral membrane ( Figure 6d–e). After this “verticularization” process, the lateral membrane can proceed to the elongation phase to acquire proper height on the Z-axis ( Figure 6f–g). Conversion of an orthogonal lateral membrane to a vertical lateral membrane can conceivably be induced by forces (along the Y-axis) acting on the X-axis of cell-cell interface ( Figure 6h and i).

## Future perspectives

Understanding how an epithelial cell forms the intercellular interface and modulates cell-cell adhesion, in two and three dimensions, would require new ways to describe the lateral membrane. Analysis tools and methodology to measure cell behaviors in two and three dimensions on the vertical axis, and with time as the fourth dimension, will be necessary to elucidate not only the biochemical and biophysical nature of the lateral membrane but also the biology that is performed by cell-cell interactions.
